# Assessment of clinical prognosis in autoimmune encephalitis: Girona score

**DOI:** 10.3389/fpsyt.2025.1447009

**Published:** 2025-01-29

**Authors:** Gary Álvarez Bravo, Giuseppe Guglielmini, Ana Quiroga Varela, Almudena Boix Lago, Ariadna Gifreu Fraixinó, Daniele Urso, Giancarlo Logroscino, Lluís Ramió-Torrentà

**Affiliations:** ^1^ Neurodegeneration and Neuroinflammation group Unitat de Neuroimmunologia i Esclerosi Múltiple Territorial Girona, Departamento de Neurologia, Hospital Universitari de Girona Dr. Josep Trueta, Girona, Spain; ^2^ Neurodegeneration and Neuroinflammation group Unitat de Neuroimmunologia i Esclerosi Múltiple Territorial Girona, Institute of Biomedical Research of Girona, Girona, Spain; ^3^ Center for Neurodegenerative Diseases and the Aging Brain, Department of Clinical Research in Neurology, University of Bari “Aldo Moro”, “Pia Fondazione Cardinale G. Panico”, Tricase, Lecce, Italy; ^4^ Department of Neurosciences, Institute of Psychiatry, Psychology and Neuroscience, King’s College London, London, United Kingdom; ^5^ Parkinson’s Foundation Centre of Excellence, King’s College Hospital, London, United Kingdom; ^6^ Department of Neurology, University of Bari Aldo Moro, Bari, Italy

**Keywords:** autoimmune encephalitis, dysautonomia, altered mental status, epilepsy, steroids

## Abstract

**Background:**

The assessment of clinical prognosis in autoimmune encephalitis: Girona (ACPE-Gi) score is a scale for evaluating the severity in the acute phase of autoimmune encephalitis (AE) and predicting the risk of disability at 3 months, measured by modified Rankin scale (mRS).

**Methods:**

Patients were strictly diagnosed with AE according to the current criteria between 1 January 2009 to 31 March 2023 at the University Hospital Dr. Josep Trueta of Girona, Catalonia, Spain. ACPE-Gi score included 14 items, and every item was scored from 0 to 3, depending on their severity with a sum ranging from 0 to 41.

**Results:**

ACPE-Gi score measured the severity in the acute phase and grouped the patients into three groups: mild (<8; 32%), moderate (8 to 15; 60%), and severe (>15; 8%). We found that the third group had a higher risk of disability compared with the first group (p = 0.035). We identified that the mean initial score was significantly higher in the group of patients who had higher mRS at 3 months compared to that in the group of patients who had a mild to moderate disability level (mRS ≤ 2) at 3 months (p = 0.023). In addition, autonomic symptoms and mental status impairment demonstrated to be independent risk factors to predict disability (p < 0.05).

**Conclusions:**

The ACPE-Gi score seems to be a reliable scale for comprehensively evaluating the severity of AE in the acute phase and predicting the risk of disability at 3 months. Dysautonomia and altered mental status predict a poorer prognosis in patients with AE.

## Introduction

Autoimmune encephalitides (AEs) are a heterogeneous group of immune-mediated disorders with subacute onset that affect the central, peripheral, and autonomic nervous systems (1). Due to this multifocal involvement, an integrative system of assessment is imperative. Scales or tools that accurately measure the severity and predict the risk of disability are still lacking in the approach of AE.

AEs are caused by different types of antibodies that not only share some clinical characteristics but also differ in their pathophysiological mechanisms and presumably in their prognosis (2). Behind these particularities, it is crucial to identify some patients at risk of disability in order to try individualized therapies that improve their functional outcomes. Currently, AEs are responsible for 20% of all types of encephalitis, and, although physicians are more aware of its detection over time, a more homogeneous approach is still needed (3).

We propose the Assessment of clinical prognosis in autoimmune encephalitis: Girona (ACPE-Gi) score to evaluate the severity of AE at diagnosis or after relapses and predict the risk of disability at 3 months. The main goal of this scale is to carry out prompt therapeutic interventions that modify the clinical course based on early and precise detection of the risk of poor functional outcomes.

Items such as seizures, memory dysfunction, psychiatric symptoms, consciousness, language problems, movement disorders, ataxia, brainstem dysfunction, pyramidal/sensory dysfunctions, neuroimaging findings, autonomic symptoms, risk of cancer association according to the type of antibodies, and recurrences were considered for developing the scale. Unlike other clinical scales, we have scored the neuroimaging, autonomic function, risk of cancer association, and recurrences because we consider these items as important characteristics for the comprehensive evaluation of AE.

The ACPE-Gi score is the first score that assesses most of clinical domains reported in the pathogenesis of AE, adding the weight of the neuroimaging. Thus, we aim to verify the internal validity of ACPE-Gi score.

## Methods

### Data source

Patients were strictly diagnosed with AE according to the current criteria at the University Hospital Dr. Josep Trueta of Girona, Catalonia-Spain.

The inclusion criteria were (1) patients aged > 16 years, (2) patients with acute/subacute AE onset, and (3) patients with at least one systemic screening for tumors.

Patients with either infectious encephalitis or from undetermined etiologies were excluded from the study. Patients with insufficient key clinical data were not considered either.

Antibodies testing were analyzed through indirect immunofluorescence testing or cell-based assays. All samples were tested at the admission.

### Standard protocol approvals, registrations, and patient consents

This study was approved by the Ethical Committee of the University Hospital Dr. Josep Trueta of Girona. A written informed consent was obtained from the patients.

All methods were performed in accordance with the relevant guidelines and regulations.

### Study populations

Patients with AE with available data from 1 January 2009 to 31 March 2023 were analyzed. All participants signed a written informed consent before the start of the study.

A total of 81 patients were enrolled, 56 of whom were excluded as they did not fulfill criteria for AE diagnosis or lack of key clinical data.

Complications during hospital stay were considered as systemic or non-neurological complications with prolonged the length of stay.

### Data collection

This study collected patients’ baseline demographics, modified Rankin scale (mRS) at admission and 3 months after treatment, treatment regimens, complications during stay in the hospital, and details of the 14 variables involved in the ACPE-Gi score at admission.

All patients with AE were scored retrospectively on the basis of their clinical features on admission (before treatment). After discharge, mRS was collected face to face at the 3-month follow-up visit by three neurologists who are experts in autoimmune encephalitis. The mRS at 3 months is performed as part of our clinical practice after every discharge from the hospital.

This is a study conducted at the Unit of Neuroimmunology and Multiple Sclerosis of Girona (UNIEMTG). Investigators are neurologists with expertise in neuroimmunology.

ACPE-Gi score was developed by Álvarez et al., to evaluate more extensively the severity of autoimmune encephalitis and identify if higher scores on admission are in higher risk of disability at 3 months measured by mRS and depending on the therapeutic strategy used.

Fourteen items were included into the proposed score (**Table 1**): seizures, memory dysfunction, psychiatric symptoms, consciousness, language problems, dyskinesia/dystonia, gait instability and ataxia, brainstem dysfunction, muscle weakness, brain and spinal cord MRI abnormalities, autonomic dysfunction, risk of cancer association depending on antibody, recurrences, and rapid progression (<15 days to bedridden). Every item was scored from 0 to 3 depending on their severity, with a sum ranging from 0 to 41. The exception was the rapidly progressive course that was scored with 2 points if present. Recurrences were scored according to the number of episodes and maximum of 3 points. A clinical relapse or recurrence was characterized as a new onset or further deterioration of a pre-existing condition.

**Table 1:** Assessment of clinical prognosis in autoimmune encephalitis: Girona score.

**Seizures**	None Controlled seizures Intractable seizures* Status epilepticus	**0** **1** **2** **3**	**Gait instability and ataxia**	Normal Mild, able to walk unassisted Moderate, assisted walking Severe, unable to walk	**0** **1** **2** **3**
**Memory dysfunction**	None Mild (does not affect daily activities) Moderate (interferes with daily activities) Severe (no recent memory or unable to communicate)	**0** **1** **2** **3**	**Brainstem dysfunction (number of symptoms)**	None Gaze paresis Tube feeding Ventilator care due to central hypoventilation	**0** **1** **2** **3**
**Psychiatric symptoms**	None Mild (no need for medical intervention because it does not affect daily activities) Moderate (need for medical intervention because it interferes with daily activities) Severe (needs continuous care or admission because of psychiatric symptoms) or unable to check	**0** **1** **2** **3**	**Muscle weakness**	Normal (Grade V) Mild (Grade IV) Moderate (Grade III) Severe (Grade II or less)	**0** **1** **2** **3**
**Consciousness**	Alert (opens eyes spontaneously) Drowsy (opens eyes to voice) Stupor (opens eyes to pain) Comatose (does not open eyes)	**0** **1** **2** **3**	**Brain and spinal cord MRI**	Normal Limbic encephalitis (uni or bilateral) Focal alterations non-Limbic encephalitis (LE) Multifocal or diffuse alterations	**0** **1** **2** **3**
**Language problem**	None Mild (slow but able to express sentences) Moderate (unable to express full sentences) Severe (unable to communicate)	**0** **1** **2** **3**	**Autonomic dysfunction (non-epileptic)**	Normal Isolated symptoms of autonomic lability (LA) More than 1 symptom suggestive of LA Paroxysmal sympathetic hyperactivity	**0** **1** **2** **3**
**Dyskinesia/dystonia**	None Mild dyskinesia (does not affect daily activities) Moderate dyskinesia (interferes with daily activities) Severe dyskinesia causing secondary medical problems	**0** **1** **2** **3**	**Cancer association**	None Low-risk antibodies Intermediate-risk antibodies High-risk antibodies	**0** **1** **2** **3**
**Rapid Progression**	No Yes	**1** **2**	**Recurrences**	First episode Recurrences*	**1** **2**

*If the recurrence occurred under the proposed therapeutic approach, look for other strategy.

List of clinical features and scores highlighted in bold. Score of clinical severity: 0 normal, 1 mild involvement, 2: moderate involvement, 3 severe involvement.

Early and timely treatment was considered as starting immunotherapy within 4 weeks of disease onset. Different therapeutic strategies, such as methylprednisolone, intravenous immunoglobulins, rituximab, and combined treatments, were administered.

We stratified the patient’s clinical condition in the acute phase using the ACPE-Gi score, as mild (<8), moderate (8–15), or severe (>15).

Good and poor prognoses were determined as mRS score ≤2 and an mRS score >2, respectively, after 3 months of follow-up.

### Statistical analysis

The collected data were analyzed using statistical software SPSS version 25. Descriptive and frequency analyses were conducted to assess the characteristics of the study population.

The correlation between the Gi score and the mRS at 3 months was initially evaluated using linear regression. A p-value of less than 0.05 was considered statistically significant.

To evaluate the effect of the score on the mRS at 3 months, patients were divided into three groups on the basis of increasing scores (<8, 8–15, and >15). Univariate analysis of the data was performed using the Kruskal–Wallis; a p-value of <0.05 was considered statistically significant.

In addition, to further assess the association between the proposed score and mRS at 3 months, a logistic regression analysis was used; a significance level of p < 0.05 was considered as the threshold for determining the statistical significance of the relationships.

We conducted an Analysis of Variance (ANOVA) to compare the mean 3-month mRS scores across different antibody types. The antibody types included seronegative, intracellular antigens, surface antigens, and others.

Every item of Gi score was analyzed independently to find out its impact on the functional prognosis, with each item being evaluated separately on the basis of the presence or absence of the disorder using the Student t-test; a p-value of <0.05 was considered statistically significant.

In another step, the patients were categorized into two groups on the basis of the 3-month prognosis (mRS < 3 and mRS ≥ 3) to assess potential significant differences in the initial mean score. Subsequently, a t-test was conducted, with a significance threshold set at p < 0.05.

## Results

### Patient characteristics

This study comprised 25 patients visited at the Unit of Neuroimmunology and Multiple Sclerosis of the University Hospital Dr. Josep Trueta of Girona. The median age at disease onset was 58 (Interquartile range (IQR): 18–79). Eighteen men (72%) were included in the study.

Three patients (12%) were diagnosed with antibody against leucine-rich glioma inactivated 1 (anti-LGI1) encephalitis, three (12%) with antibody against Glutamic acid decarboxylase (anti-GAD) encephalitis, two (8%) with antibody against contactin-associated protein-like 2 (anti-CASPR2) encephalitis, two (8%) with encephalomyelitis antibody against glial fibrillar acidic protein (anti-GFAP), two (8%) with anti-Hu encephalitis, one (4%) with anti-Ri encephalitis, one (4%) with antibody against Sry-like high-mobility group box 1 (anti-SOX1) encephalitis, one (4%) with SREAT (steroid-responsive encephalopathy associated with autoimmune thyroiditis), one (4%) with antibody against immunoglobulin-like cell adhesion molecule 5 (IgLON5) antibody encephalitis, one (4%) with antibody against Gamma-aminobutyric acid receptor, type A receptor (GABAAR) antibody encephalitis, one (4%) with antibody against N-methyl D-aspartate receptor (anti-NMDAR) encephalitis, one (4%) with antibody against the CV2/collapsin response mediator protein 5 (CV2) antibody encephalitis, and six (24%) with seronegative autoimmune encephalitis (**Table 2**).

**Table 2:** Patient profiles: demographics, mRS, and encephalitis antibodies.

Variable	N	Details						
**Age of onset (years)**	25	Mean (SD): 54.0 (17.73) Range (min–max): 18–79						
**Male sex n (%)**	24	18 (72)						
**Type of antibodies n (%)**	24	**Seronegative:** 6 (24) **Neuronal surface:** 8 (32) **Intracellular:** 10 (40) **Others:** 1 (4)						
**Acute-phase mRS, n (%)**	24	**0** 0 (0)	**1** 0 (0)	**2** 5 (20.8)	**3** 10 (41.7)	**4** 3 (12.5)	**5** 6 (25.0)	**6** 0 (0)
**3-Month mRS, n (%)**	25	**0** 0 (0)	**1** 6 (24.0)	**2** 8 (32.0)	**3** 2 (8.0)	**4** 0 (0)	**5** 0 (0)	**6** 4 (16.0)

List of clinical variables and scores highlighted in bold. Scores of modified Rankin scale (mRS): mRS 0 = No symptoms at all; mRS 1 = No significant disability despite symptoms; able to carry out all usual duties and activities; mRS 2 = Slight disability; unable to carry out all previous activities, but able to look after own affairs without assistance; mRS 3 = Moderate disability; requiring some help, but able to walk without assistance; mRS 4 = Moderately severe disability; unable to walk and attend to bodily needs without assistance; mRS 5 = Severe disability; bedridden, incontinent and requiring constant nursing care and attention; mRS 6 = dead.

### Risk of poor prognosis

**Figure 1:**
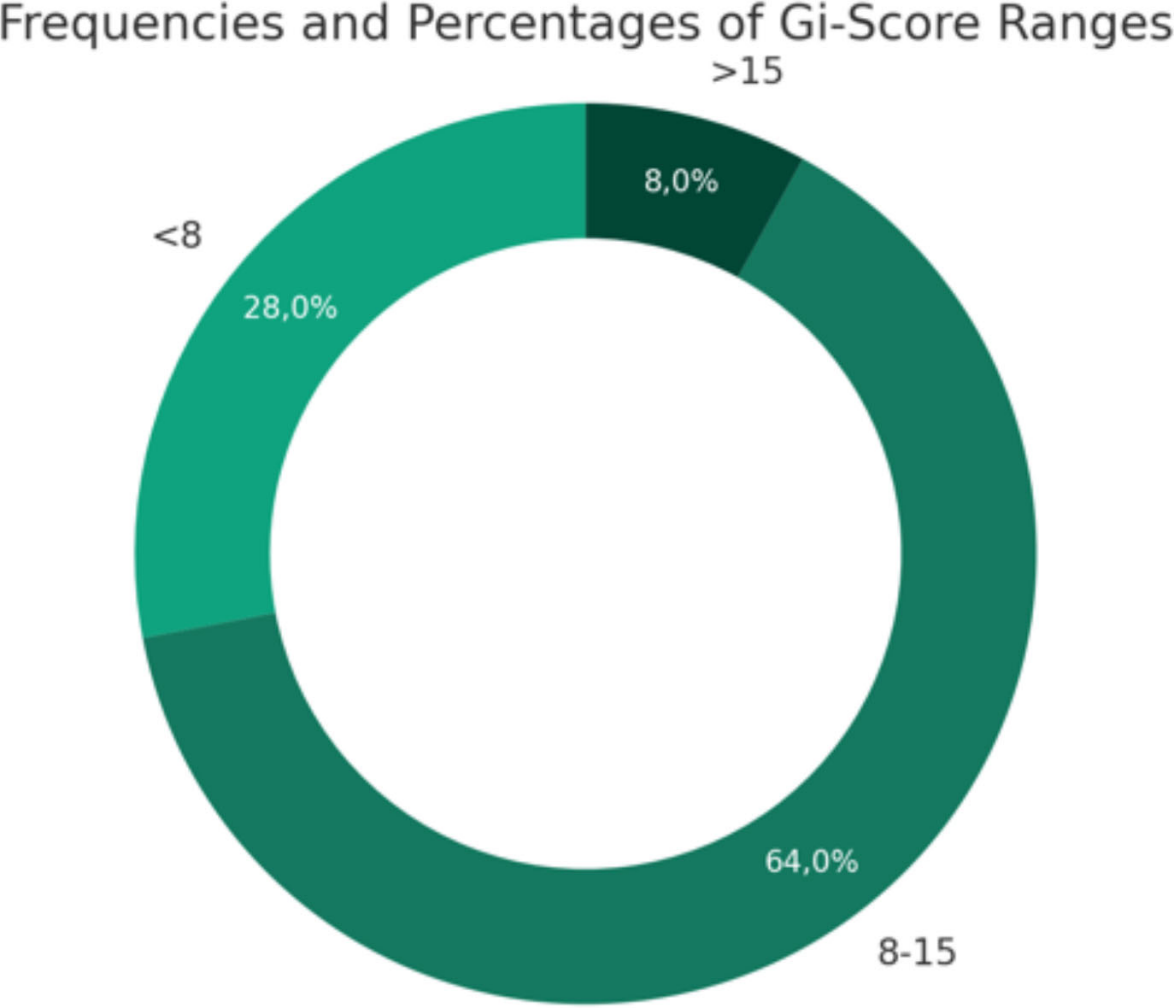
Frequencies and percentages of Gi-score ranges.

Based on the degree of clinical involvement in the acute phase, 7 (28%) patients had a mild degree, 16 (64%) had a moderate degree, and 2 (8%) had a severe degree (**Figure 1**).

The mean score during the acute phase was 10.48 (IQR: 4–18; median: 10.00).

The clinical characteristics of our sample for each considered item are expressed as frequency and percentage in **Table 3**.

**Table 3:** Detailed clinical characteristics of the sample based on Gi-score items.

Score		0 Points	1 Point	2 Points	3 Points
**Seizure**		No crisis (%)	Controlled crisis (%)	Uncontrolled crisis (%)	Status epilepticus (%)
	25	12 (48)	7 (28)	2 (8)	4 (16)
**Memory**		No alterations (%)	Does not affect ADLs (%)	Affects ADLs (%)	No recent memory or unable to communicate (%)
	25	8 (32)	7 (28)	8 (32)	2 (8)
**Psychiatric**		No alterations (%)	No treatment, does not affect ADLs (%)	Treatment, affects ADLs (%)	Requires continuous care or hospital admission (%)
	25	12 (48)	9 (36)	3 (12)	1 (4)
**Consciousness**		No alterations (%)	Somnolent (opens eyes to voice) (%)	Stuporous (opens eyes to pain) (%)	Does not open eyes (%)
	25	13 (52)	9 (36)	2 (8)	1 (4)
**Language**		No alterations (%)	Mild (altered fluency) (%)	Moderate (unable to express complete sentences) (%)	Severe (unable to communicate) (%)
	25	10 (40)	8 (32)	7 (28)	0 (0)
**Dyskinesia/dystonia**		No alterations (%)	Does not affect ADLs (%)	Affects ADLs (%)	Causes secondary medical problems (%)
	25	20 (80)	1 (4)	2 (8)	2 (8)
**Gait instability**		No alterations (%)	Mild (can walk without assistance) (%)	Moderate (walks with assistance) (%)	Severe (unable to walk) (%)
	25	12 (48)	10 (40)	2 (8)	1 (4)
**Brainstem**		No alterations (%)	Gaze paresis (%)	Nasogastric tube (%)	Mechanical ventilation for central hypoventilation (%)
	25	17 (68)	4 (16)	4 (16)	0 (0)
**Weakness**		Normal: MRC, 5 (%)	Mild: MRC, 4 (%)	Moderate: MRC, 3 (%)	Severe: MRC, 2 or less (%)
	25	18 (72)	6 (24)	1 (4)	0 (0)
**MRI**		No alterations (%)	Limbic encephalitis (uni- or bilateral) (%)	Focal alterations (not limbic encephalitis) (%)	Alterations multifocal or diffuse (%)
	25	3 (12)	11 (44)	10 (40)	1 (4)
**Autonomic**		No alterations (%)	Isolated symptom (%)	More than one symptom (%)	Paroxysmal sympathetic hyperactivity (%)
	25	17 (68)	4 (16)	2 (8)	2 (8)
**Risk cancer**		No (%)	Low risk antibodies (%)	Moderate risk antibodies (%)	High-risk antibodies (%)
	25	9 (36)	7 (28)	4 (16)	5 (20)
**Recurrence**			First episode (%)	One recurrence (%)	Two or more recurrence (%)
	25		23 (92)	2 (8)	0 (0)
**Rapid progression**		No (%)	Yes (%)		
	25	13 (52)	12 (48)		

List of clinical features highlighted in bold.

**Figure 2:**
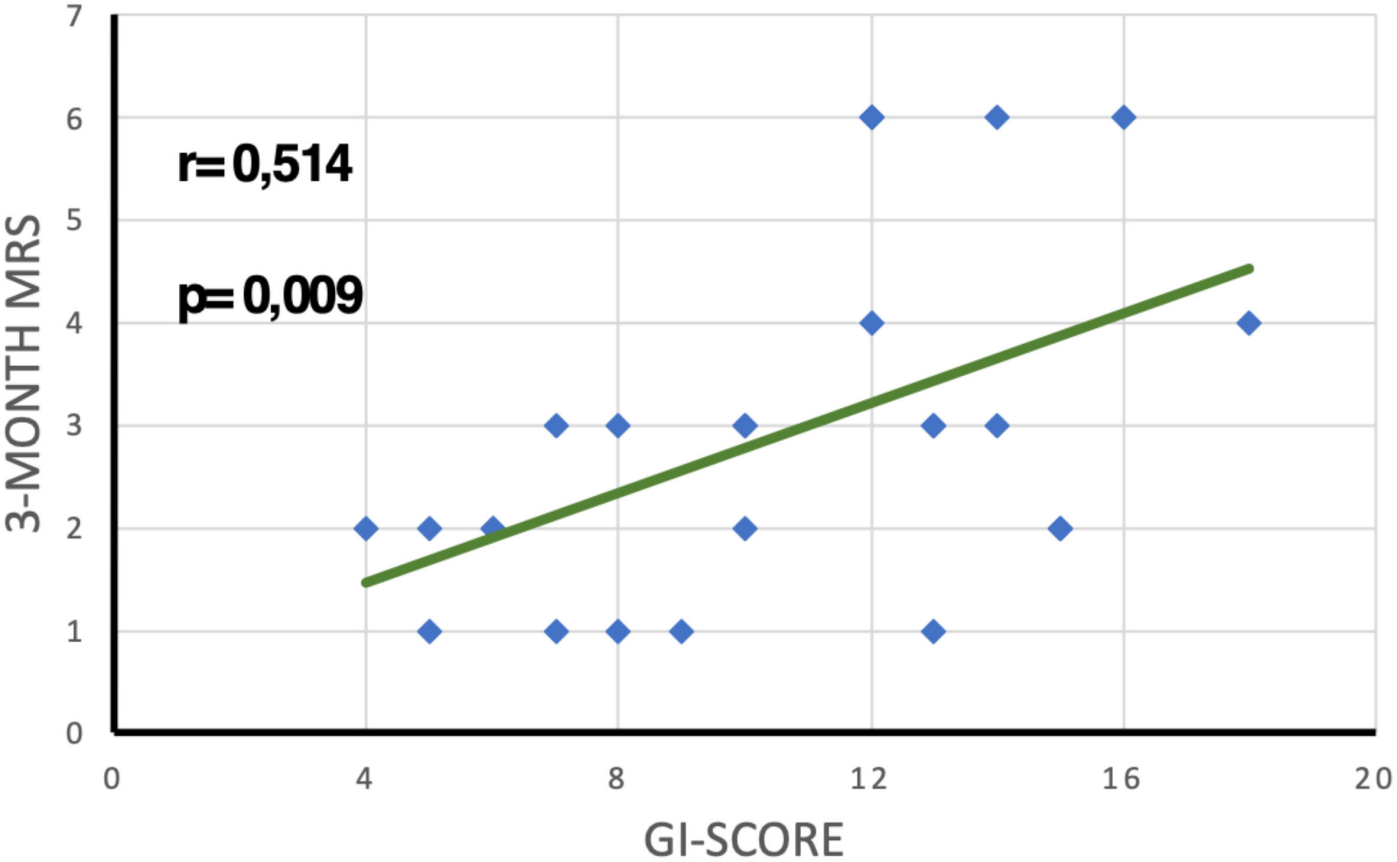
Linear regression analysis showing the relationship between GI score and 3-month mRS.

As a preliminary step, we aimed to elucidate the nature of the relationship between the initial phase score (Gi score) and the 3-month mRS of patients through a linear regression test. The outcome indicated a moderate positive correlation (r = 0.514) which was statistically significant (p < 0.05) (**Figure 2**).

**Figure 3:**
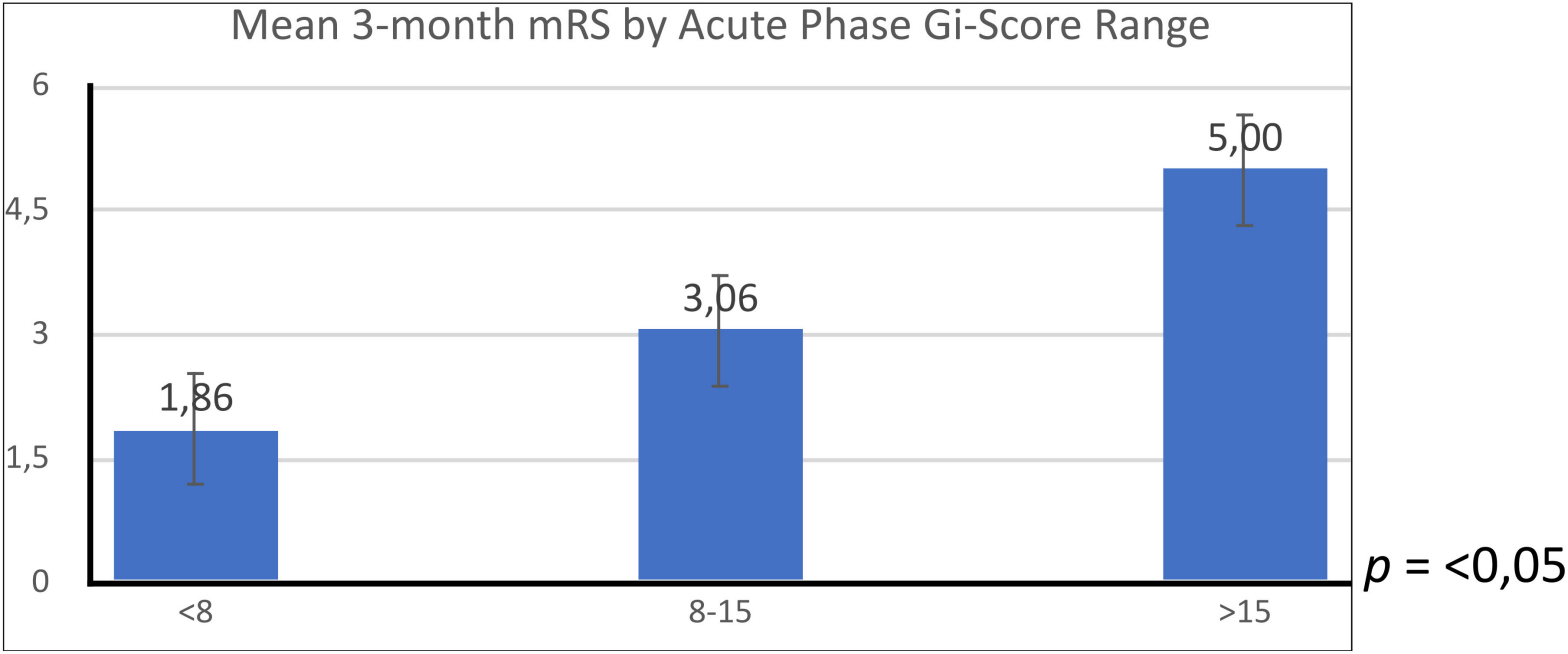
Comparison of mean 3-month modified Rankin scale (mRS) scores across different acute-phase GI-score ranges.

Subsequently, we compared the average 3-month mRS by stratifying patients according to acute-phase Gi-score ranges using the Kruskal–Wallis test (**Figure 3**).

The Kruskal–Wallis H test yielded a statistic of 6.48 with 2 degrees of freedom. The asymptotic significance of the test was 0.05, indicating that there are statistically significant differences between the groups in terms of their 3-month mRS scores when grouped by the acute-phase Gi-score range.

**Figure 4:**
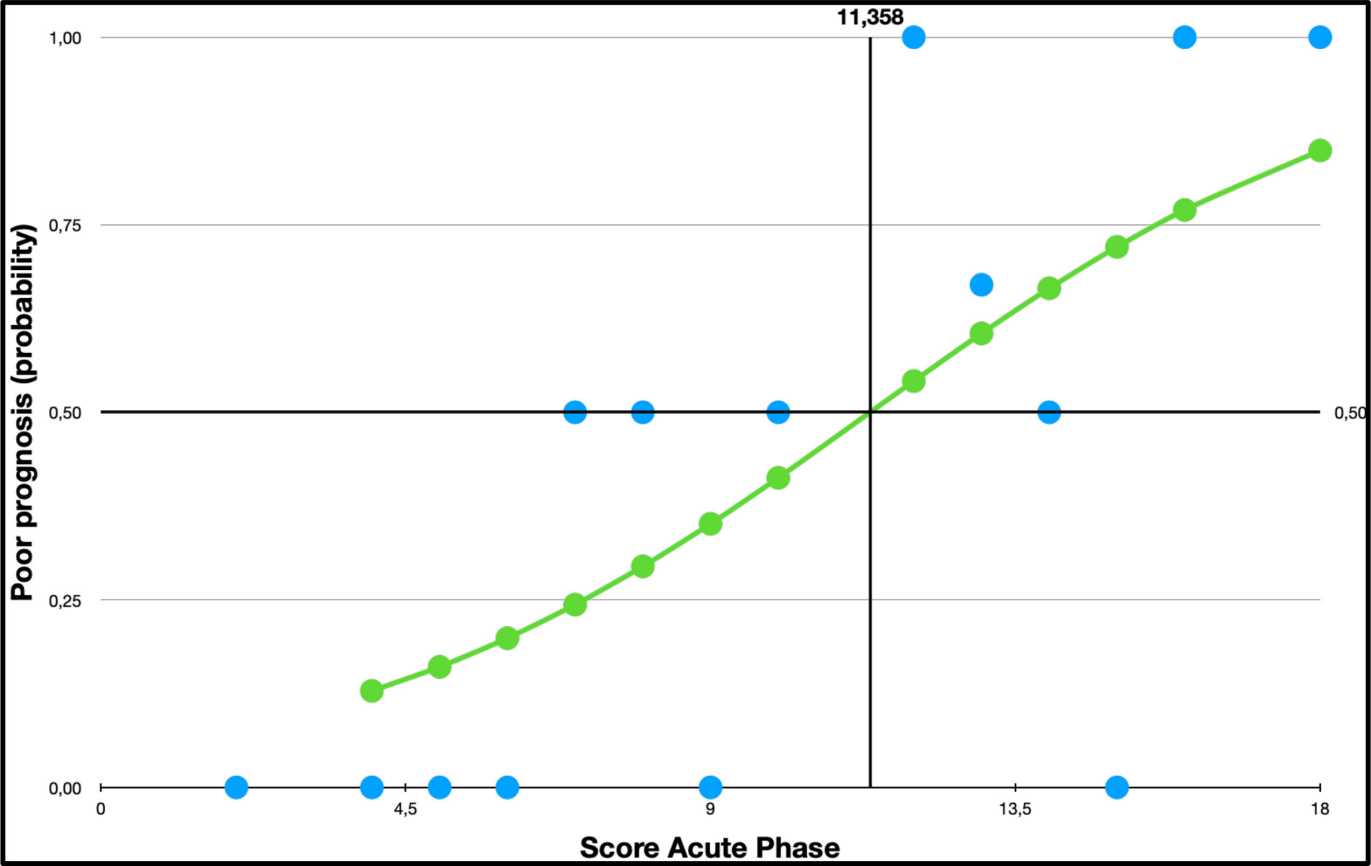
Logistic regression probability curve with data points:mRS by Gi score.

The logistic regression analysis showed a significant association between the initial clinical score and the risk of having an mRS >2 at 3 months (*p =* 0.05). We calculated a 50% of probabilities of having a mRS > 2 at 3 months when the score was of 11.36. Each point increased in the score indicated a higher likelihood of having an mRS >2 at 3 months. The odds ratio calculated was 1.29 (**Figure 4**).

### Analysis according to type of antibodies

**Figure 5:**
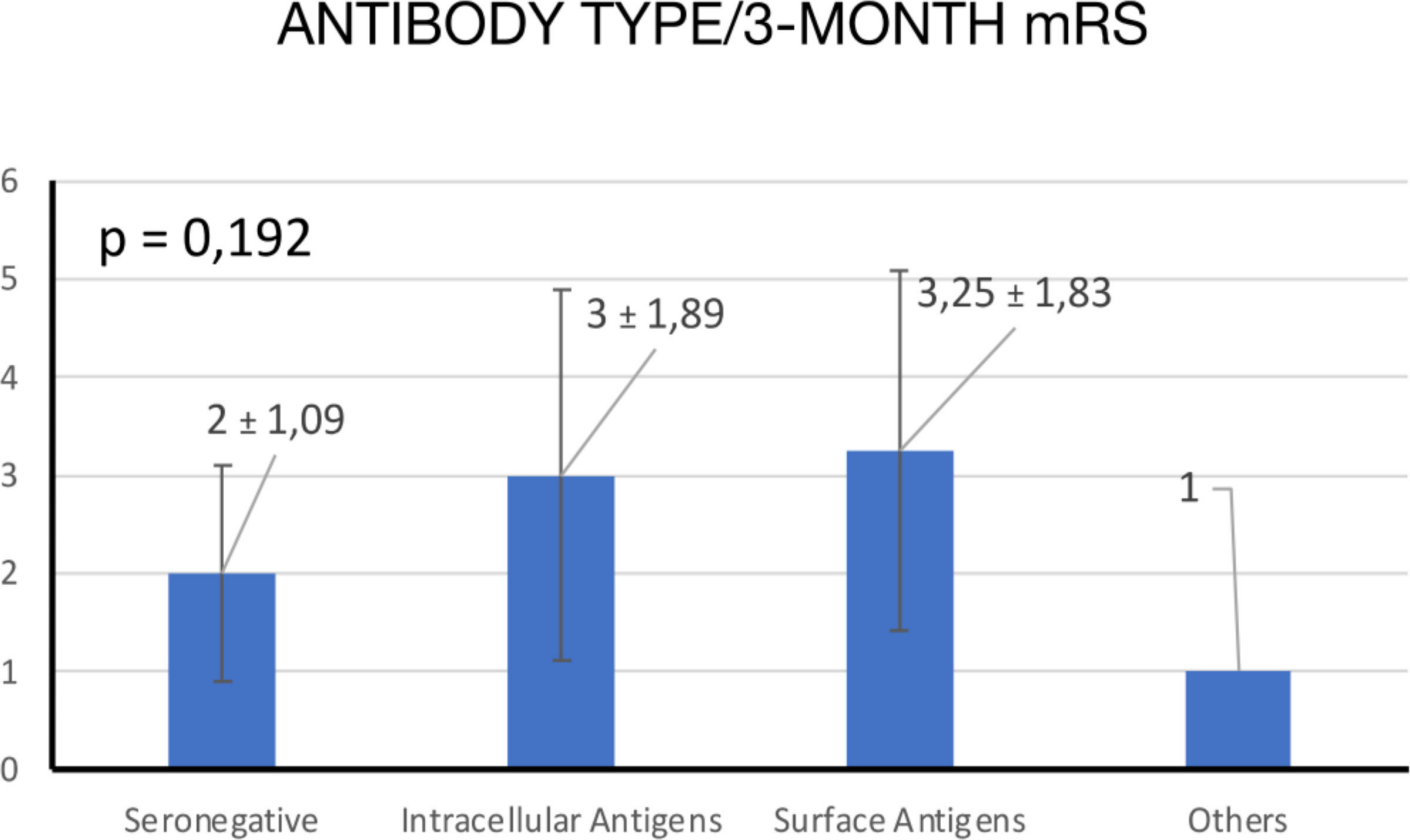
ANOVA comparison of mean 3-month mRS scores by antibody type.

Nineteen (76%) patients were seropositive for any type of antibodies. Of them, 10 had antibodies against intracellular antigens, 8 had antibodies against surface antigens, and 1 had thyroid peroxidase antibodies. The latter was not attributed to either of the two previous categories. We did not find statistically significant differences between the groups according to the type of antibody in prognosis at 3 months measured by mRS (*p =* 0.192) (**Figure 5**).

### Analysis based on mRS

Eleven (44%) patients had mRS >2 at 3 months. Of them, four (16%) patients died.

**Figure 6:**
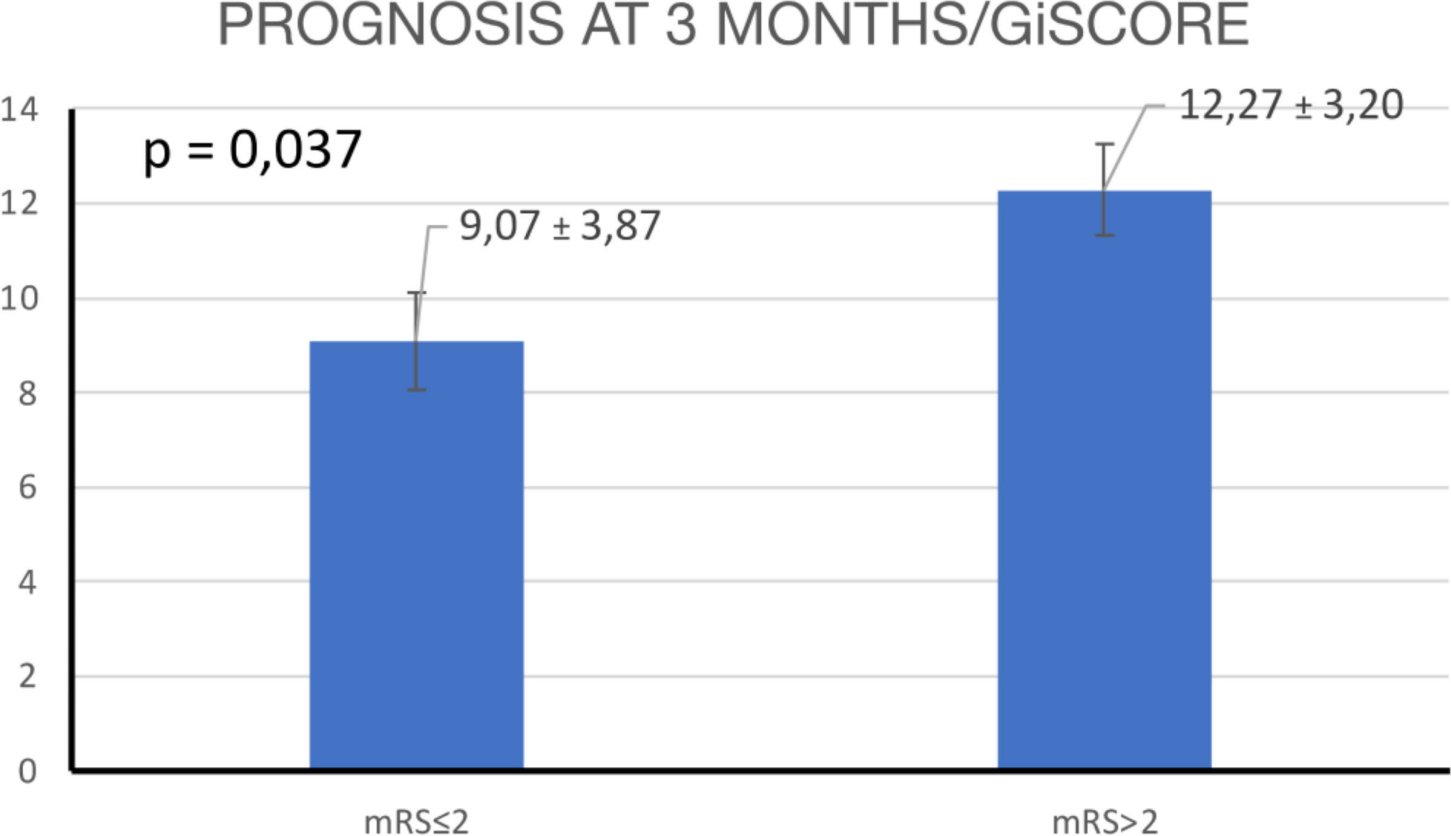
T-test comparison of mean Gi score at 3 months based on mRS categories.

The t-test showed significant differences in the mean initial scores between patients with a worse prognosis (12.27 ± 3.2) and those who showed a better prognosis (9.07 ± 3.87) at 3 months (*p =* 0.037) (**Figure 6**).

### Analysis based on isolated variables

**Figure 7:**
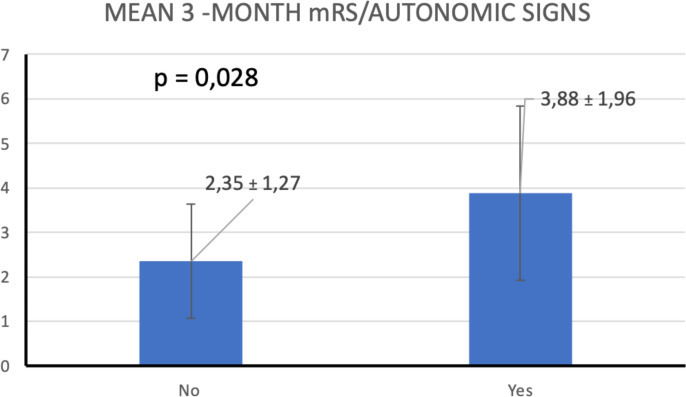
T-test comparison of mean 3-month mRS scores by autonomic signs.

**Figure 8:**
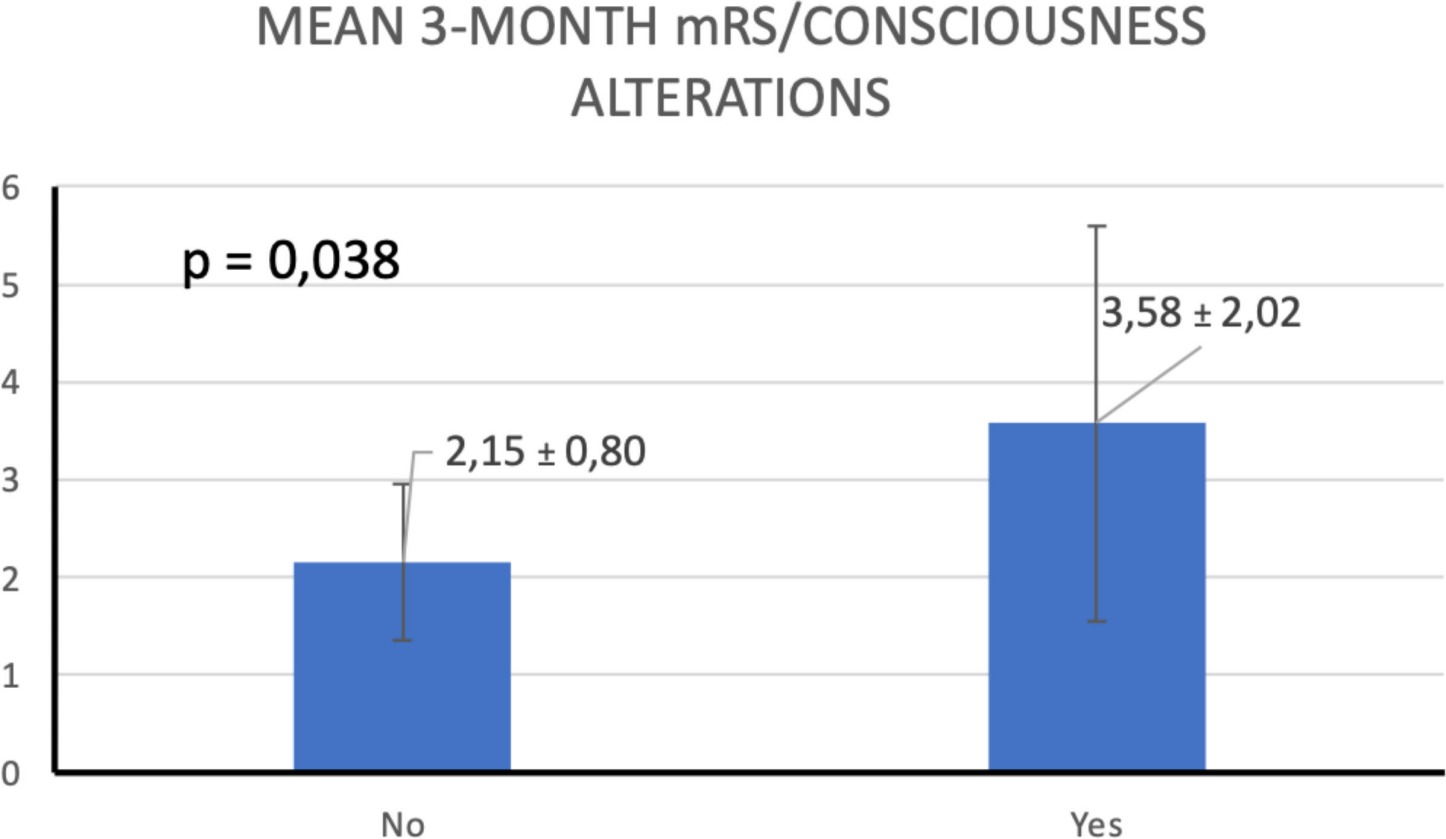
T-test comparison of mean 3-month mRS scores by consciousness alterations.

Seventeen patients (68%) had short-term memory impairment. This was the most common clinical feature. Eight patients (32%) had some degree of autonomic dysfunction. This group had a higher risk of mRS >2 at 3 months (*p =* 0.028) compared with patients with no autonomic symptoms. (**Figure 7**). Altered mental status was the other symptom that strongly predict a higher mRS at 3 months (p = 0.038) (**Figure 8**). No other clinical or radiological features were found to be a reliable predictor of disability (**Table 4**).

**Table 4:** Comparison of mean with standard deviation for the presence of each variable of the Gi score.

	No	Yes	P-value
**Seizure**	2.92 ± 1.68	2.77 ± 1.69	0.83
**Memory**	3.38 ± 2.26	2.59 ± 1.28	0.38
**Psychiatric**	3.08 ± 2.02	2.62 ± 1.26	0.50
**Consciousness**	2.15 ± 0.80	3.58 ± 2.02	0.038
**Language**	2.70 ± 1.49	2.93 ± 1.79	0.74
**Dyskinesia/dystonia**	2.85 ± 1.63	2.80 ± 1.92	0.95
**Gait instability**	2.42 ± 1.44	3.23 ± 1.79	0.24
**Brainstem**	2.53 ± 1.50	3.50 ± 1.85	0.97
**Weakness**	3.11 ± 1.74	2.14 ± 1.21	0.19
**MRI**	2.00 ± 1.00	2.95 ± 1.70	0.10
**Autonomic**	2.35 ± 1.27	3.88 ± 1.96	0.026
**Risk cancer**	2.00 ± 0.87	3.31 ± 1.81	0.054
**Recurrence**	2.70 ± 1.58	4.50 ± 2.12	0.14
**Rapid progression**	2.75 ± 1.71	2.92 ± 1.66	0.80

List of clinical features highlighted in bold.

An underlying tumor was detected in four patients. Three patients were seropositive and had been classified as in higher risk of cancer association (n = 2) and in intermediate risk of cancer association (n = 1), respectively. Antibodies with lower risk of cancer association were the type most commonly detected.

A total of 88% of patients had brain MRI abnormalities. Limbic encephalitis was the pattern most commonly observed (44%). However, there were no statistically significant differences in the average 3-month mRS between patients with and without MRI abnormalities (p = 0.36).

Two patients had a relapsing course, but this clinical presentation was not a good predictor of loss of physical independence (p = 0.14).

Twelve patients (48%) had a rapidly progressive course. However, no statistically significant differences were observed in the average 3-month mRS between patients with rapid acute-phase progression and those without (p = 0.80).

Two patients had clinical complications that prolonged the length of hospital stay. Both patients had ventilator-associated pneumonia.

### Analysis of poor prognosis risk based on the type of immunotherapy

All patients were treated during the acute phase. A total of 56% received methylprednisolone exclusively, whereas the remaining 44% received methylprednisolone along with another immunotherapy (Intravenous immunoglobulin (IVIG) or rituximab). There were not statistically differences in prognosis based on the therapeutic strategy used during the acute phase.

## Discussion

Autoimmune encephalitides are a group of disorders with subacute onset that affect the central, peripheral, and autonomic nervous systems (1). Due to the multifocal involvement, there is a marked heterogeneity in the clinical features observed in AE (1, 2). Distinctive symptoms such as psychiatric manifestations, epilepsy, memory loss, and/or altered mental status constitute the clinical core for the diagnosis (3). Although the diagnosis remains rare, in the last years, there have been an increasing number of scientific reports exposing uncommon symptoms that have allowed to expand the clinical spectrum (4, 5). Every year, new antibodies are discovered and posed as biomarkers for distinguishing specific type of AE (6). These discoveries increase the awareness among clinicians and have eased the detection of AE over time.

Antibodies against neuronal cell-surface proteins, ion channels, or intracellular receptors have been reported as the main causes of AE. The advent of specific autoantibodies has greatly provided a better understanding of pathogenesis of AE. In spite of these autoantibodies having excellent roles as diagnostic markers, some of them have not been shown to have direct roles in neuronal dysfunction (7).

The wide range of clinical characteristics probably derives from the heterogeneity of autoantibodies and their implicit and particular pathophysiological mechanisms that give rise to the need to develop a comprehensive system for the evaluation of autoimmune encephalitis in the acute setting.

There is a need of validated clinical scales to assess the autoimmune encephalitis, despite that they might be a useful tool to evaluate the acute attack and predict the risk of disability derived from the initial injury. For instance, some symptoms including cognitive dysfunction and epilepsy are widely reported in the different case series of AE during the acute phase, notwithstanding their persistence as sequelae is not yet fully elucidated and their impact on activities of daily living is scarcely studied (8).

Some scales have been proposed for several research groups, among them, the Clinical Assessment Scale in Autoimmune Encephalitis (CASE) validated for Chinese patients. It measures the severity of the autoimmune encephalitis at time of diagnosis and correlate the score with modified Rankin score at 12 months, attempting to predict the disability after the acute phase. The CASE authors proposed the scale as a suitable tool for the comprehensive assessment of Chinese patients with autoimmune encephalitis, which may help clinicians to select the appropriate intervention and estimate the disease severity and prognosis (9).

Through the ACPE-Gi score, we propose a scale that integrates different items that have not been considered in other tools. In order to broaden knowledge in AE assessment, we added some variables such as autonomic symptoms, risk of cancer association, MRI features, recurrences, and rapid progression. To our knowledge, there are no other scales that predict the prognosis of AE based on the type of clinic-radiological variables.

The aforementioned clinical features were added on the basis of our clinical observations and after exhaustively reviewing some clinical reports describing some specific symptoms of AD.

The added variables were considered as important features within the integral and holistic evaluation of the patients with autoimmune encephalitis by the authors.

Classical clinical features, such as the alteration of consciousness included into the CASE, as well as into the ACPE-Gi, demonstrated a strong association to predict worse functional prognosis at 3 months (10).

Dysautonomia is a common clinical manifestation of AE. It has mainly been reported in association to anti-NMDAR encephalitis. Autonomic dysfunction can occur at different stages of the disease, sometimes even throughout the disease, and may even increase the risk of ICU admission (10, 11). Our results agreed with the conclusions of Yan et al. that showed that patients with autonomic dysfunction had a higher incidence of decreased consciousness over the course of the disease and a worse prognosis (12). Patients with AE with autonomic dysfunction have also a higher incidence of pulmonary infection and abnormal liver function and these complications can unfavorably impact on their prognosis (13); nevertheless, in our study, the demonstrated risk of disability associated to autonomic dysfunction was independent of systemic complications.

Recurrence was the other clinical feature added to our clinical assessment. Some AEs have a relapsing phenotype, and, depending on the symptoms, the relapse severity could seriously affect the patient prognosis (14). Nevertheless, there is no consensus to attributing the recurrences as a good predictor for poor prognosis (15). In our study, we could not demonstrate that patients who experienced relapses had a poorer prognosis compared with those with monophasic disease, although we still consider that this feature should be taken into account in the holistic AE evaluation.

Some autoimmune encephalitis develop a rapidly progressive encephalopathy due to an exaggerated autoimmune response directed against the brain parenchyma. A rapid onset within 2 weeks is associated with higher rates of morbidity (16) and presumably with higher risk of disability.

Up to 55% of patients with AE with a rapid-onset can be admitted to the neurocritical care unit (16).

Symptoms including status epilepticus and paroxysmal sympathetic hyperactivity require prompt specific interventions and immunotherapy in the intensive care unit (17). In our study, a rapid onset was not associated to risk of disability at 3 months, but a higher number of patients and further studies are necessary to clarify this hypothesis.

To our knowledge, this is the first study that attempts to give weight to neuroimaging in the assessment of disability risk in AE.

Limbic encephalitis is the most consistent radiological pattern in autoimmune encephalitis (18). However, non-limbic focal alterations can also be observed in the neocortex, striatum, hindbrain, spine, and peripheral nervous system depending on some specific antibody profile (19, 20). We had hypothesized that there would be a higher risk of disability in patients with greater brain volume involved during the acute phase of AD, but this theory could not be demonstrated in our study. Nonetheless, the role of the neuroimaging to predict the risk of poor prognosis is a pathway to be explored for additional researches.

The risk of cancer association according to the type of antibody is other novelty introduced in our score. Despite that antibodies do not have an absolute association with cancer, the authors considered this variable as an independent item to be evaluated, because the clinical recognition of antibodies in higher risk of cancer association determines a specific assessment mainly focused on the intensive search for an underlying malignancy (21). Although this variable was not directly associated with a worse prognosis in our study, we propose seeking individualized strategies aimed at optimizing therapies, considering the type of antibody, as an essential part of the comprehensive evaluation of AEs.

Of added features in ACPE-Gi score, only dysautonomia showed a correlation statistically significant to predict a poor prognosis at 3 months measured by mRS. Three patients died because of paroxysmal sympathetic hyperactivity. Although dysautonomia is recognized as a serious complication in the acute phase of some AE, it is barely reported in the case series, and the underlying pathophysiological mechanisms are not fully understood (13).

Autoimmune encephalitides can have different dynamics in the clinical course. So, patients with anti-NMDAR encephalitis present a higher median mRS at 3 months compared to patients with anti-LGI1/CASPR2 encephalitis, although 70% of them recovered mRS <2 at 12 months. Some authors suggest that this disparity is explained by the different types of immunotherapy used during the acute phase (22). Our results reflect with statistical significance that a higher score is a good predictor of disability at 3 months measured by modified Rankin score regardless of the type of antibodies and the immunotherapy used during the acute phase.

We observed several limitations in our study. Firstly, this was a retrospective study, and a larger prospective study should be considered to validate our results. Second, there was no homogeneity in types of immunotherapy used during the acute attack; thus, this disparity could influence on the final outcomes. Third, the small sample size collected in one center, and fourth, our results were measured in 3 months as we promote an early detection to make early interventions that modify the clinical evolution. Nevertheless, we are aware of some patients can achieve good functional outcomes later than this time (22).

In conclusion, ACPE-Gi score might be a valuable tool for assessing the extent of the initial injury or after each relapse in AE and predicting the risk of disability measured by mRS at 3 months and based on symptoms such as dysautonomia and altered mental status. In the future, the validation of this scale could guide clinical decisions, notwithstanding that further studies are needed to determine the true utility of the ACPE-Gi score.

## Data Availability

The raw data supporting the conclusions of this article will be made available by the authors, without undue reservation.
