# Supplementary material for: Assessment of clinical prognosis in autoimmune encephalitis: Girona score
Source: Front Psychiatry. 2025 Jan 29;16:1447009. doi: 10.3389/fpsyt.2025.1447009 (PMC11814168; doi:10.3389/fpsyt.2025.1447009)
Supplement: Supplementary file 1 [file Table1.docx]

**Supplementary material**

**Table of patients included in ACPE-Gi according to neural auto-antibody (NAA)**

| **Antibody/Status** | **Number of Patients (Percentage)** |
| --- | --- |
| **Anti-LGI1 encephalitis** | 3 (12%) |
| **Anti-GAD encephalitis** | 3 (12%) |
| **Anti-CASPR2 encephalitis** | 2 (8%) |
| **Encephalomyelitis anti-GFAP** | 2 (8%) |
| **Anti-Hu encephalitis** | 2 (8%) |
| **Anti-Ri encephalitis** | 1 (4%) |
| **Anti-SOX1 encephalitis** | 1 (4%) |
| **SREAT (steroid-responsive encephalopathy)** | 1 (4%) |
| **IgLON5 antibody encephalitis** | 1 (4%) |
| **GABAAR antibody encephalitis** | 1 (4%) |
| **Anti-NMDAR encephalitis** | 1 (4%) |
| **CV2 antibody encephalitis** | 1 (4%) |
| **Seronegative autoimmune encephalitis** | 6 (24%) |
